# Application of natural polysaccharides and their novel dosage forms in gynecological cancers: therapeutic implications from the diversity potential of natural compounds

**DOI:** 10.3389/fphar.2023.1195104

**Published:** 2023-06-13

**Authors:** Yi Li, Chuanlong Zhang, Lu Feng, Qian Shen, Fudong Liu, Xiaochen Jiang, Bo Pang

**Affiliations:** ^1^ Guang’anmen Hospital, China Academy of Chinese Medical Sciences, Beijing, China; ^2^ College of Acupuncture-Moxibustion and Tuina, Shandong University of Traditional Chinese Medicine, Jinan, China; ^3^ International Medical Department of Guang’anmen Hospital, China Academy of Chinese Medical Sciences, Beijing, China

**Keywords:** gynecological cancers, polysaccharides, cervical cancer, endometrial cancer, natural compound, ovarian cancer

## Abstract

Cancer is one of the most lethal diseases. Globally, the number of cancers is nearly 10 million per year. Gynecological cancers (for instance, ovarian, cervical, and endometrial), relying on hidden diseases, misdiagnoses, and high recurrence rates, have seriously affected women’s health. Traditional chemotherapy, hormone therapy, targeted therapy, and immunotherapy effectively improve the prognosis of gynecological cancer patients. However, with the emergence of adverse reactions and drug resistance, leading to the occurrence of complications and poor compliance of patients, we have to focus on the new treatment direction of gynecological cancers. Because of the potential effects of natural drugs in regulating immune function, protecting against oxidative damage, and improving the energy metabolism of the body, natural compounds represented by polysaccharides have also attracted extensive attention in recent years. More and more studies have shown that polysaccharides are effective in the treatment of various tumors and in reducing the burden of metastasis. In this review, we focus on the positive role of natural polysaccharides in the treatment of gynecologic cancer, the molecular mechanisms, and the available evidence, and discuss the potential use of new dosage forms derived from polysaccharides in gynecologic cancer. This study covers the most comprehensive discussion on applying natural polysaccharides and their novel preparations in gynecological cancers. By providing complete and valuable sources of information, we hope to promote more effective treatment solutions for clinical diagnosis and treatment of gynecological cancers.

## 1 Introduction

Globally, more than 1 million women are diagnosed with female reproductive tract cancer each year, mainly covering cervical, ovarian, endometrial, fallopian tube, vulva, and vaginal malignancies (Siegel et al., 2019). The three most common gynecological cancers are cervical, ovarian, and endometrial cancer. Data shows that in 2020, 60,4127 new cervical cancer, 417,367 cases of endometrial cancer, and 31,3959 cases of ovarian cancer (Sung et al., 2021), its high recurrence, and high mortality, seriously affected women’s health in the world (including Southern Africa). In addition, the potential ‘Financial Toxicity’ brought by gynecological cancers has also caused massive growth in global medical expenses (Mariotto et al., 2020). In the United States, the estimated costs for gynecological cancers treatment in 2018 were $ 1543.9 billion for cervical cancer, US $ 2.945.7 billion for endometrial cancer, and ovarian cancer 5.8626 billion US dollars, which will grow sharply by 2030, causing a vast and increasing growth burden (Carrera et al., 2018).

Affected by age, obesity, metabolism, and reproductive factors, gynecological cancers always develop in a direction (Crocker et al., 2021), that is, difficult to predict. The reaction rate of traditional therapy is meager, and treating gynecological cancers in traditional standard conventional treatment (for instance, surgery, chemotherapy, and radiotherapy) has been as complicated as starting a damaged ‘machine.’ In recent years, molecular biology technology has led to a new boom in developing new tumor drugs. Poly ADP-ribose polymerase inhibitors, antibody-drug conjugate, and immunotherapy have brought new gynecological cancer treatments and improved disease prognosis. The breakthroughs, but most of these drugs are still underway, and the mechanism of their effects remains to be further studied.

Research by Newman et al. shows that the research and development of about 75% of anticancer drugs are from natural products (Newman and Cragg, 2016). Moreover, evidence shows that a variety of natural compounds (for instance, polysaccharides, polyphenols, alkaloids, triterpenoids, quinones, steroids, and flavonoids) have unique cellular selectivity, high efficiency, low prices, and side reactions, which makes it to find new anti-gynecological cancers drugs with potential treatment effects from natural compounds a hot spot for research (Liu et al., 2020; Pan et al., 2023). Many biological properties of natural polysaccharides have been discovered, including immune regulation (Sunila et al., 2011), antitumor (Ahmad, 2020), antiviral (Ray et al., 2021), antioxidant (Zhang et al., 2021), anti-inflammatory (Hou et al., 2020), blood glucose and blood lipid regulation (Zaporozhets and Besednova, 2016), and it is also a beneficial auxiliary and chemical prevention agent for gynecological cancers (Deng et al., 2020; Lyu et al., 2020).

This article reviews the positive effects, molecular mechanisms, and evidence of natural polysaccharides in gynecological cancer treatment and then introduces them. It then introduces the relationship between natural polysaccharides and new dosage types and drug transportation systems of polysaccharides, microcapsules, lipids, nanoparticles, and other new dosage types. Although most of the current research is limited to cells and animal experiments, this reviews the research progress of natural anti-gynecological cancers polysaccharides in nearly 20 years, providing detailed and diversified sources of information which will help anti-gynecological cancers natural drug-screening and research and development work.

## 2 Natural polysaccharides and their structure-activity relationship

Natural polysaccharides are composed of monosaccharides connected to the covalent glycoside. The skeleton structure is diverse and complicated and has powerful biological information-carrying capabilities. Studies have found that the biological activity of natural polysaccharides is regulated by their monosaccharide composition, molecular weight, glycosidic bond type, and sulfate content, which are important factors affecting the conformation and spatial structure of polysaccharides chain structure (Jiao et al., 2016). In terms of monosaccharide composition, polysaccharides composed of galactose, mouse sugar, mannose, and Arabian sugar have high biological activity (Kralovec et al., 2007). In terms of molecular weight, evidence shows that low molecular weight polysaccharides may have better physical activity conditioning (Li et al., 2013). The polysaccharide glycoside bonds are mainly composed of α-(1→6)-D, α-(1→4)-D, and β-(1→4)-D. Polysaccharides represented by the α-spiral structure, such as rosa rugosa polysaccharide, Cucurbita moschata polysaccharide, and cistanche deserticola polysaccharide, show solid biological functions (Yin et al., 2019a). In addition, researchers regarded the content of sulfate as an essential indicator of polysaccharides in recent years. According to reports, the tension between sulfate groups can lead to the relative expansion of the polysaccharide chain, and this relatively high chain rigidity enhances polysaccharide activity under certain conditions (Liao et al., 2013). Bimalendu Ray et al. (2020) found that polysaccharide sulfuric can carry a negative charge within a specific pH value range, affecting biomolecules moving in ain positive change direction. It is related to the amount of sulfate content involving the combination of cells (Dinoro et al., 2019).

In the study of natural polysaccharides, we have not found a higher polysaccharide antitumor activity composed of monosaccharides. Still, in terms of molecular weight, some studies have shown the difference in antitumor activity. In studying Lycium Barbarum Polysaccharide LBPA4 and LBP-p8, researchers such as Min (Zhang et al., 2013) found that the relative molecular mass of LBPA4 was 10.2kda, and the relative molecular mass of LBP-p8 was 6.5 × 103 kda, but the former showed resistance to resistance. Cancer activity, while the latter cannot change the cell cycle of human liver cancer cells and the concentration of Ca^2+^ in the cytoplasm without anticancer indicates that low molecular weight polysaccharides are better antitumor drug candidates. Most polysaccharides with antitumor activity used to be the main chain, mainly based on β-(1→3)-D-glucan. It seems that this unique structure is an essential point of polysaccharide antitumor. However, subsequent studies have shown that this understanding is one-sided, such as Pachyman almost does not have antitumor activity, but thee. However, the overall view of β-(1→3)-D-glucan backbone is still a universal structure of natural polysaccharides antitumor (Rioux et al., 2010; Jing et al., 2022). In addition to the influence of the intervention of the main chain structure, the polysaccharide chain also affects the biological activity of the polysaccharides. Studies have shown that polysaccharides with antitumor activity have at least (1→6)-β branch (Wang et al., 2019). By comparison of the synthetic alpha-1,6 branched glycogen, Ryoyama et al. (2004) found that reducing the degree of antitumor and immune cells can weaken polysaccharides. In terms of glycoside bonds, most of the polysaccharides with antitumor effects are composed of similar alkaline glucan structures and different types of glycoside bonds, especially the polysaccharide antitumor activity connected by β-(1→3)-glycoside and β-(1→6)-glycoside is the best activity, which may be related to the residue of the β-glycoside connection to exert the effect of antitumor, anti-inflammatory, and immune regulation (Tzianabos, 2000; Chen et al., 2022). In terms of polysaccharide chain conformations, among many space structures such as single screw, double helix, triple helix, random coil, and aggregate, the triple helix is the most active spatial structure. Studies have confirmed the hydrophilicity of the triple helix surface, which group can play a practical antitumor effect by regulating immune activity (Chen et al., 2014).

## 3 The role of natural polysaccharides in gynecological cancers

**FIGURE 1:**
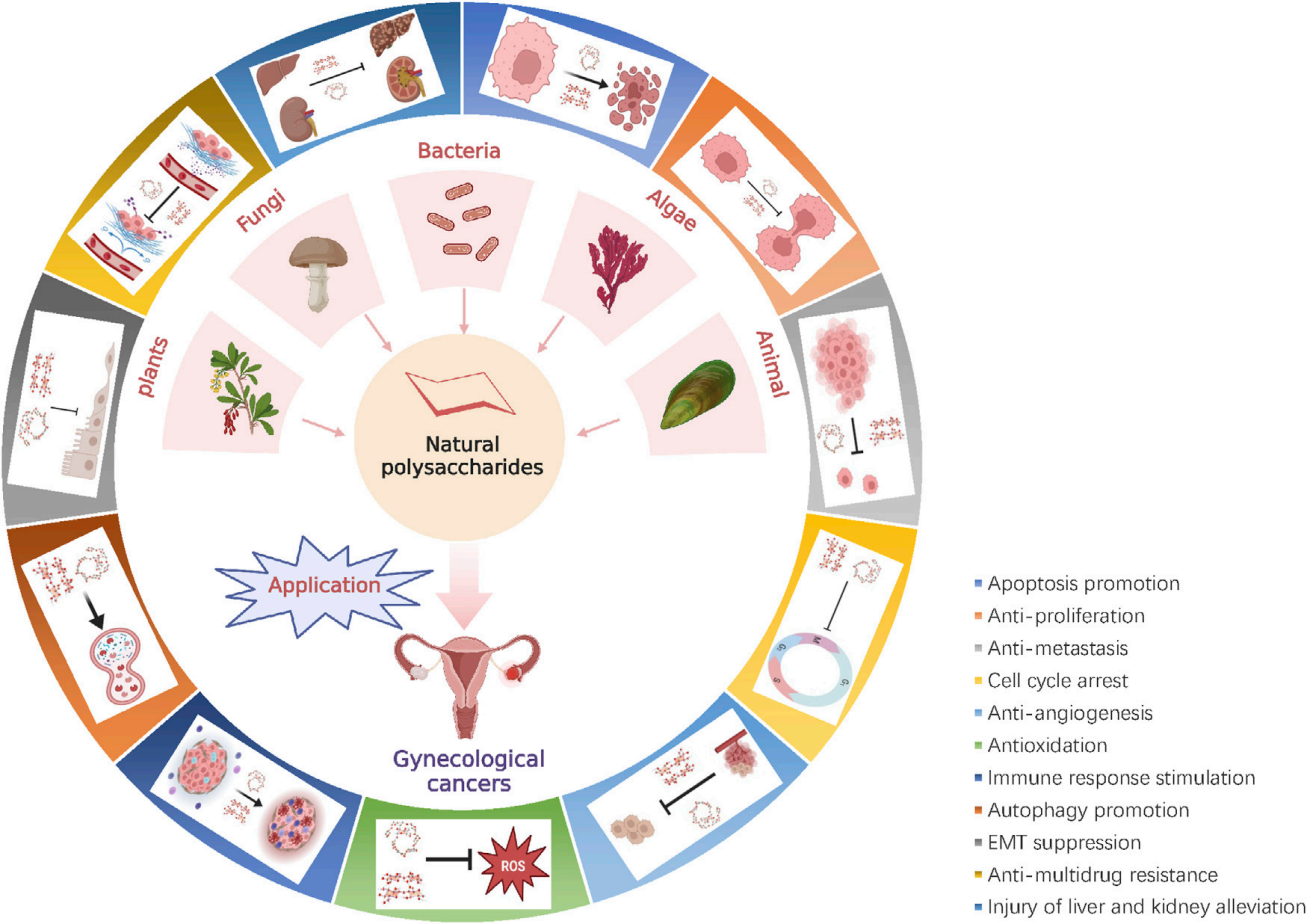
Representative source species of natural polysaccharides and their anti-gynecological cancers mechanism. Natural polysaccharides can potentially improve the prognosis in gynecologic cancer, by inhibiting tumor cell proliferation, promoting tumor cell apoptosis, inhibiting tumor cell invasion and migration, antioxidant, anti-inflammatory, activating the immune suppression, EMT, promoting autophagy, anti-angiogenesis, blocking the cell cycle, regulation of signaling pathways.

Natural polysaccharides are wide sources. More than 300 biological active polysaccharides are currently separated from natural products. They are widely distributed among the high plants, animals, fungi, bacteria, and algae in nature (Yu et al., 2018). Healthy food and fruits, vegetables (including edible fungi), and marine-derived animals and plants are very rich in natural polysaccharides, especially in kiwi, pumpkin, shiitake mushrooms, seaweed, mussels, and other foods, as well as Poria, Yuanzhi, astragalus, wolfberry and other traditional Chinese medicine (TCM) in the material. In 1969, Chihara et al. (1969) reported the natural polysaccharide β-(1→3) glucan lentinan (LNT) with antitumor activity for the first time. In 1988, Shimizu et al. (1988) proved for the first time that LNT could increase the occurrence of cervical cancer patients with IL-2 cells in pelvic lymph nodes. Subsequently, more and more studies have confirmed that natural polysaccharides are beneficial auxiliary agents and chemical prevention agents for gynecological cancers (Wong et al., 1994; Zeng et al., 2019). Natural polysaccharides can reduce risk factors such as HPV infection, obesity, and inflammation during the beginning of gynecological cancers and progress (Derby et al., 2018; Sauruk da Silva et al., 2021). At the same time, the ROS levels of tumor cells can be induced, leading to mitochondrial destruction and DNA damage, enlarging the apoptosis-level reaction, and then inhibiting the proliferation of tumor cells and promoting apoptosis (Kobayashi et al., 2005). The detailed mechanism of natural polysaccharide against gynecological tumors is shown in Figure 1.

### 3.1 Polysaccharides and cervical cancer

Cervical cancer is the most malignant tumor in gynecological cancers, and 13% of patients with cervical cancer are diagnosed in the advanced stage. Patients with metastatic cervical cancer such as metastatic cervical cancer are poor, and the 5-year survival rate is only 16.5% (Li et al., 2016). At present, surgery and chemotherapy are still the basic means of cervical cancer treatment, but it is undeniable that these standard therapies will cause some damage to vaginal and ovarian function (LaVigne et al., 2017). As the research on natural products continues to deepen, polysaccharides from high plants, fungi, bacteria and seaweed, and animals are found in treating cervical cancer. Studies have shown that polysaccharides can play an anti-cervical cancer effect by improving the immune organ’s index, alleviating oxidation stress, blocking the tumor cell cycle, and triggering tumor apoptosis. In Table 1, we list the detailed results of natural polysaccharides anti cervical cancer.

**TABLE 1:** Compilation of experimental data related to the protective effect of natural polysaccharides anti-cervical cancer.

Classification	Compound/Extract	Abbreviation of polysaccharides	Cell Line/Animal model	Mechanism and effect	Ref
Polysaccharides of higher plants	*Portulaca oleracea L.* polysaccharide	POL-P3b	Kunming mice (18–22 g)	Inhibit Akt, NF-κB activity and Bcl-2 expression in intestinal dendritic cells; Stimulate the protection of TLR4-PI3K/AKT-NF-κB signaling pathway on apoptosis of intestinal dendritic cells	Zhao et al. (2019)
	*Solanum nigrum L.* polysaccharide	SNL-P	U14 cells; Kunming mice (aged 6–8 weeks, 18–22 g)	Reduce the level of TNF-α in serum; Upregulate the expression level of Bax; Downregulate the expression of Bcl-2 and mutant p53 gene; Trigger apoptosis of tumor cells	Li et al. (2007)
	*Patrinia heterophylla Bunge* polysaccharide	PHB-P1	U14 cells; Kunming mice (aged 6–8 weeks, 18–22 g)	Increase the number of GO/G1 phase cells and reduce the number of S phase cells; Upregulate the expression of p53 and Bax; Inhibit the expression of Bcl-2	Lu et al. (2009)
	*Atractylodes lancea (Thunb.) DC.* polysaccharide	ACPS-1	HeLa cells	Inhibit cell proliferation; Block the cell cycle	Xu et al. (2016)
	*Lycium barbarum L.* polysaccharide	LBP	HeLa cells	Change cell cycle distribution; Induce apoptosis to inhibit the proliferation	Zhu and Zhang (2013)
	*Garden Cress* polysaccharide	GCP	HeLa cells	Reduce the production of free radicals	Alkahtani et al. (2020)
	*Acanthopanax senticosus* polysaccharide	ASPS	U14 cells	Increase the content of IL-2 and IL-12 in serum; Increase the index of immune organs such as spleen and thymus; Regulate immunity; Reverse apoptosis of immune cells	Meng et al. (2018)
			Kunming mice (aged 6–7 weeks, 18–22 g)		
	*Angelica sinensis (Oliv.) Diels* polysaccharide	APS-1d	HeLa cells; athymic BALB/c nude mice (aged 6 weeks)	Improve the expression of Bax and Bak; Reduce the expression of anti-apoptosis proteins Bcl-2 and Bcl-XL; Reduce the mitochondrial membrane potential; Stimulate the mitochondrial apoptosis pathway	Cao et al. (2010)
Polysaccharides from fungi	*Hansfordia sinuosae* extracellular polysaccharide	HPA	HeLa cells	Increase ROS level; Reduce mitochondrial membrane potential; Increase caspase-3 expression; Inducing apoptosis	Li et al. (2018a)
	Enzymatic hydrolysis of *Ganoderma lucidum* polysaccharide	EGLP	U14 cells; Kunming mice (aged 6–8 weeks, 18–22 g)	Protect immune organs; Improve the activity of antioxidant enzymes; Increase the expression of Bax and cleaved caspase-3; Decrease the expression of Bcl-2 and COX-2 to inhibit tumor proliferation	Kong et al. (2019)
	Polysaccharides extracted from *wild Russula griseocarnosa*	PRG1-1	HeLa cells; SiHa cells	Increase the production of lactate dehydrogenase and ROS; Increase the apoptosis rate; Increase the cytoplasmic expression of PARP, cytochrome c and caspase-3; Reduce the expression of cytochrome c in mitochondria; Prevent DNA repair of tumor cells; Aggravate the cumulative damage of DNA; Increase the production of LDH and ROS.	Yuan et al. (2017), Liu et al. (2018)
	*Lentinus edodes* polysaccharide	LEP1	HeLa cells	Increase Bax/Bcl-2 ratio; Reduce mitochondrial membrane potential; Promote cytochrome c release from mitochondria to cytoplasm; Activate caspase-9 and caspase-3; Cleave PARP.	Ya (2017)
Polysaccharides from bacteria	Extracellular polymeric substances	EPS	HeLa cells	Relieve oxidative stress; Reduce inflammatory response	Xiao et al. (2018)
	*Laminaria japonica* sulfate polysaccharide	LJSP	U14 cells; Kunming mice (aged 6–8 weeks, 18–22 g)	Improve immune function; Increase the Bax expression; Reduce the Bcl-2 expression; Increase the Bcl-2/Bax ratio; Induce tumor cell apoptosis	Zhai et al. (2014)

#### 3.1.1 Polysaccharides of higher plants

*Portulaca oleracea L.* is a kind of natural medicine commonly used in Chinese medicine, considered to have the effect of clearing heat and poisoning. According to reports, the water-soluble polysaccharide POL-P3b extracted from *Portulaca oleracea L.* is evaluated with antioxidant and immune regulatory activity and can enhance immune function by removing free radicals accumulated by the body (Manzo, 1988). Tumor cells evade immune recognition and attack by modifying their surface antigens, changing the local microenvironment, and then suppressing the function of immune cells (Rabinovich et al., 2007). Zhao et al. (2019) discovered that the intestinal DCs carrying the U14 mouse and intestinal DCs will have increased apoptosis, and Pol-P3b can effectively protect the intestinal DCs from apoptosis. The protection method of Pol-P3b anti-cervical cancer has dosage dependence. The specific mechanism is related to the stimulation of TLR4/PI3K/AKT-NF-κB signal pathway stimulation to the protection of the apoptosis of intestinal DCs. Another polysaccharide, PSP, composed of glucose and galactose, is also found in gynecological cancer treatment. YouGuo et al. (2009) have confirmed that PSP has a strong oxidation damage protection effect, can inhibit H_2_O_2_ -induced red blood cell hemolysis phenomenon, protect red blood cell function, and enhance thymic cell proliferation in concentration dependence.

*Solanum nigrum L.* has been clinically applied for several centuries. It has previously been proven to have extensive biological activity and has the unique effects of antitumor, anti-inflammatory, and diuretic (Jain et al., 2011). To evaluate the antitumor effect of *Solanum nigrum L.* polysaccharides, Li et al. (2007), Li et al. (2010a) separated the crude polysaccharides from the *Solanum nigrum L.* The study found that SNL-P had a significant growth inhibitory effect on the U14 cells. Over the years, eliminating cancer cells around apoptosis and effectively eliminating cancer cells has always been an essential window for clinical cancer treatment (Carneiro and El-Deiry, 2020). Wong (2011) found that SNL-P could reduce the serum TNF-α level in U14 cervical cancer tumor-bearing mice. Many tumor cells in the SNL-P intervention group showed different degrees of nuclear chromatin clumping and cell contour convolution, which indicated that the tumor cells had undergone apoptosis. At the same time, the study also found that apoptosis is closely related to the expression of SNL-P participation, raising the Bax gene and lowering the Bcl-2 gene and mutation P53 gene expression. In addition, *in vivo* experiments showed that SNL-P had no obvious pathological effects on the kidney and liver, and was a safe and effective natural adjuvant for the treatment of cervical cancer.

*Patricia heterophylla Bunge* (PHB) is a valuable and popular natural drug. PHB-P1 is a kind of natural polysaccharide extracted from the lost sauce, and Lu et al. (2010) reported that PHB-P1 has a good body anti-cervical cancer effect. Through the weight of a mouse tumor body that was treated with different concentrations of PHB-P1, the author observed that as the PHB-P1 treatment concentration increased, the number of apoptosis cervical cancer cells in mice increased significantly. At the same time, more cells remained in the G2/M phase, and the proportion of cells in the S phase decreased, which has been proven to be closely related to the expression of BCL-2 in tumor tissue and improving the level of serum alkaline phosphatase (Lu et al., 2009). Many studies have shown that serum lactate dehydrogenase participates in the growth of tumors in tumors (Kviecinski et al., 2008). It is worth noting that PHB-P1 can reduce serum lactic acid dehydrogenase activity, indicating that the anti-cervical cancer mechanism may be more complicated and still needs to be explored in the future.

ACPS-1 is a new type of polysaccharide with a molecular weight of 11.2 KDa from *Atractylodes lancea (Thunb.) DC.*, which contains (2→1)-linked-d-Fruf residues linear backbone structure. It has been proven to suppress the proliferation of HeLa cells *in vitro*. ACPS-1 can significantly block the cell cycle while promoting the apoptosis of HeLa cells (Chow et al., 2003; Xu et al., 2016). The surprising discovery of this experiment was that the researchers found that ACPS-1 also had an inhibitory effect on SK-OV-3 cells, and the impact of promoting the apoptosis of tumors was very significant, indicating that ACPS-1 may be a potential candidate for various gynecological cancers treatment essence.

*Lepidium Sativum L.*, also known as Garden Cress (GC), is a cross-flower plant native to West Asia and Egypt. It is not only used in cooking but also in traditional folk medicine worldwide. The plant chemical analysis of GC shows that it contains many natural ingredients such as flavonoids, alkaloids, phenols, and polysaccharides, which can be used as an anti-inflammatory analgesic, antitumor, and antioxidants (Painuli et al., 2022). Garden Cress polysaccharide (GCP) has the potential to inhibit the growth of HeLa cells *in vitro*. When the dosage of GCP is 500 μg/ml, GCP shows higher anti-tumor activity, and its potential anti-mutagenic effect is achieved by its antioxidant activity and then reducing the production of free radicals (Alkahtani et al., 2020). Unfortunately, in the published original research, the study of antitumor internal antitumor of GCP was ignored. Therefore, while having the potential to be a natural alternative to chemical anticancer drugs, it is worth emphasizing that the clinical study of GCP is still immature and great caution must be taken from the laboratory to any clinical application.

As a common natural polysaccharide, *acanthopanax senticosus* polysaccharides (ASPS) are a dietary supplement widely used to treat diseases such as tumors, diabetes, gout, and hepatitis, rarely reported adverse reactions after ASPS intake (Park et al., 2006; Chen et al., 2011; Fu et al., 2012). Tumor cells secrete immunosuppressive factors, induce immune disorders and immune effects cell abnormalities, and inhibit the host’s immune function in the micro-ring (Ahn et al., 2015). ASPS can improve the thymus and spleen index of U14 solid tumor mice, which is the standard solution in cancer therapy of immunity and reverse immune cell apoptosis (Meng et al., 2018). In addition, ASPS can also increase the contents of IL-2 and IL-12 in the mice group. As a multi-effect immune factor resisting cervical cancer, IL-2 is secreted by innate killing cells, macrophages, and auxiliary T lymphocytes (Valle-Mendiola et al., 2016). Macrophages and dendritic cells secrete IL-12, recognized as an antitumor and multi-functional cytokine that causes metastasis (Lasek et al., 1997). This study shows that ASPS is a potential biological reaction regulator for the clinical treatment of cervical cancer.

*Angelica sinensis (Oliv.) Diels* is an umbrella-shaped plant. It was an essential Chinese medicine for gynecological diseases as early as thousands of years ago. The polysaccharides in angelica are abundant, and they are considered the main contributor to the role of angelica. Studies have found that angelic pose polysaccharides can activate congenital and acquired immune systems, enhance the body’s immune function, and control tumor growth and metastasis (Kim et al., 2018). APS-1d is a new type of polysaccharide, that is, separated from *Angelica sinensis (Oliv.) Diels* that are separated from (1,4)-a-D-glucopyranosyl (Glip), which can inhibit the proliferation of cervical cancer cells *in vitro* concentration and dependence (Cao et al., 2010). Specifically, the antitumor effects involved in APS-1d are related to regulating the Bcl-2 family protein, increasing the cell soluble cell pigment c level, regulating mitochondria potential, and blocking the cell cycle. The researchers confirmed that the APS-1d had a good anti-cervical cancer effect in this study. The antitumor effect result was confirmed and established in HeLa cells’ internal and *in vitro* experiments.

#### 3.1.2 Polysaccharides from fungi

Humans have used mushrooms as food and drugs for thousands of years. Natural biological active substances in mushrooms are a reliable nutrition and drug source that benefits human health (Wasser, 2002). It is estimated that the number of mushrooms on the Earth is more than 1,500, of which about 10% are well known (Panda et al., 2022), but less than 1% are used for drug use. Mushroom contains a large number of polysaccharides with antitumor and immune stimulus characteristics. It is a natural treasure trove developed by new antitumor new pharmaceutical products. In Asian countries such as China, South Korea, and Japan, more and more mushroom derivatives are reported to be applied to the clinic (Wasser, 2014).

*Wild Russula griseocarnosa* is an edible mushroom native to southeast China. Residents call it ‘Dahongjun,’ from which PRG is a kind of crude polysaccharide extracted. Yuan et al. (2017) reported the activity of HeLa and Siha cells in vitro-human cervical cancer cells. Unfortunately, the study did not clarify the detailed mechanism of the in vitro-body anti-human cervical cancer cells. With the deepening of research, Liu et al. (2018) isolated and purified PRG1-1 from crude polysaccharide of *Wild Russula griseocarnosa*, and reported the cytotoxic effect of PRG1-1 on the cytotoxicity of cervical cancer *in vitro*. The study found that PRG1-1 can reduce the vitality of HeLa cells and SiHa cells by increasing the content of lactate dehydrogenase and active oxygen and promoting its apoptosis. As an enzyme that repairs DNA, PARP is a crucial regulator for DNA damage reactions and maintaining replication stability binding to damaged DNA in DNA fracture sites (Kang et al., 2020; Slade, 2020). Studies have shown that PRG1-1 can improve the level of cracking PARP through dose dependence, thereby preventing tumor cell DNA repair and exacerbating cumulative damage. LEP1 is a natural polysaccharide extracted from the precious edible bacteria *Lentinus edodes*. It can inhibit the value-added of cervical cancer cells by increasing the Bax/Bcl-2 ratio and interference mitochular membrane potential (Ya, 2017). Among the mice carrying HeLa cells, LEP1 can induce apoptosis by cracking PARP in HeLa cells, basically the same as the anti-cervical cancer mechanism of PRG1-1.

*Ganoderma lucidum* is native to eastern Asia and is a well-known medicinal fungus. Today, researchers have separated and identified a variety of biologically active substances, such as polysaccharides, alkaloids (Xu et al., 2011), and gradually developed them for the clinical treatment of diseases. *Ganoderma lucidum* polysaccharide (GLP) is a natural product of *Ganoderma lucidum* and has been proven effective in various tumor treatments. Researchers such as Kong et al. (2019) compared the effects of GLP and enzymatic hydrolysis of *Ganoderma lucidum* polysaccharide (EGLP). They found that both polysaccharides can increase the expression of Bax and cleaved caspase-3, and reduce the expression of BCL-2 and Cox-2 to inhibit the growth of tumors. Compared with cyclopensions, GLP and EGLP can improve oxidation stimulation, protect immune organs, and have better antitumor and lower side effects. The thymus produces T cells to participate in the host’s adaptive immunity, and the spleen maintains immune stability. Both are essential immune organs of the body. It is worth noticing that EGLP can improve the spleen and thymus index of ruminoma mice, enhance the activity of antioxidant enzymes, and inhibit the progress of cervical cancer. In addition, EGLP has a better role in accelerating U14 cell apoptosis and inhibiting tumor growth than GLP, which shows that after enzymatic disintegration, GLP anti-cervical cancer has a more substantial effect. Another interesting study is that Zhu et al. (2019) combined cisplatin therapy for clinical cervical cancer, which results in show that GLP can reduce the nephrotoxicity and neurotoxicity of cisplatin and accelerate the apoptosis of tumor cells. Qiu et al. (2018a) confirmed that the folic acid conjugated-Auricularia auricular polysaccharide-cisplatin complex could better act on cervical cancer, reduce cisplatin damage to the kidneys, and provide new directions for the study of folic acid-targeted polysaccharides. These results can help us understand the mechanism of polysaccharide antitumor and their effects and accelerate the development of new cervical cancer treatment drugs.

Ocean fungi and bacteria have formed unique metabolites in adapting to the adaptation of marine salinity, temperature, and pressure. Recently many natural metabolites separated from marine fungi and bacteria have shown antitumor, antiviral, and immune regulatory activity in in vitro experiments (Mayer and Gustafson, 2006; Lee et al., 2013). HPA is an extracellular polysaccharide extracted from the marine fungus *Hansfordia sinuosae.* Gao et al. (2018) used HeLa and MCF-7 cells to prove that HPA uses cytotoxicity in dose dependence. The fluorescence test shows that tumor cells after HPA treatment have cell contraction and fracture, showing typical cell forms. Studies have shown that HPA induces the apoptosis of cervical cancer cells by increasing caspase-3 expression in HeLa cells to reduce mitochondrial membrane potential. Extracellular polymeric substances (EPS) is an extracellular polysaccharide separated from the *thraustochytrium Striatum*. The experiments by Xiao et al. (2018) showed that EPS could produce strong cytotoxic effects on HeLa cells, relieve oxidative stress and reduce the inflammatory response, and is a promising bioactive compound for the treatment of cervical cancer. Although the experimental results of marine microorganisms showed that HPA and EPS had the potential anti-cervical cancer significance, it should be emphasized that the lack of *in vivo* experiments makes their safety not well poorly evaluated. Their antitumor mechanisms still need to be further studied in detail, and any extrapolation to the clinical application must be cautious.

#### 3.1.3 Polysaccharides from algae

In the past 10 years, people have maintained a great interest in the research on the antitumor of seaweed. In Asian countries, seaweed has been considered an essential food and drug source (Yang et al., 2010). The content of polysaccharides in seaweed is wealthy, accounting for about 50% of the dry weight, which is the storage library of natural polysaccharides (Jiao et al., 2011). LJSP is the first time that Zhai et al. (2014) isolated a sulfated polysaccharide from *Laminaria japonica*, an edible brown seaweed, which was confirmed to have anti-cervical cancer effects *in vitro* and *in vivo* **.** The internal results show that LJSP has almost no liver and kidney toxicity. It can induce the apoptosis of transplantation tumor tissue by increasing the BAX/BCL-2 to inhibit the growth of cervical cancer. LJSP improves the immune function of mice by enhancing the spleen and thymus index of the U14 cells. Ulvan is an acidized polysaccharide separated from the green seaweed *Ulva Lactuca*. Thanh et al. (2016) confirmed that has significant cytotoxicity to HeLa cells. Unfortunately, although the researchers have verified Ulvan’s antitumor results, the original research does not provide complete details of the Ulvan anti-cervicalvical cancer mechanism, which makes its clinical application prospects unclear.

### 3.2 Polysaccharides and endometrial cancer

Endometrial cancer has a high rate among menopausal and perimenopausal women, accounting for 4.5% of women’s new cancer cases, which is the second only to female reproductive tract malignant tumors after cervical cancer (Sung et al., 2021). Currently, endometrial cancer is mainly based on the comprehensive treatment of postoperative radiotherapy and chemotherapy. Compared with most gynecological cancers, endometrial cancer can be diagnosed and treated early, but there is a considerable prognosis difference between their historical types. Non-estrogen-dependent endometrium cancer (type II) tends to be even in early treatment. In recurrence, and some patients have metastasis during diagnosis, this makes the therapy of treating endometrial cancer far more tricky than we think (Amant et al., 2005). Natural products are potential supplements for endometrial cancer treatment. There is currently less research on polysaccharides for endometrial cancer, and most of them are concentrated in China. Still, more and more data show that polysaccharides can exert anti-endometrial cancer effects by activating the mitochondrial apoptosis signaling pathway, increasing the immune organ index, and arresting the tumor cell cycle. In Table 2, we list the detailed results of natural polysaccharides for anti-endometrial carcinoma.

**TABLE 2:** Compilation of experimental data related to the protective effect of natural polysaccharides anti-endometrial carcinoma.

Classification	Compound/Extract	Abbreviation of polysaccharides	Cell Line/Animal model	Mechanism and effect	References
Polysaccharides of higher plants	polysaccharide of *Atractylodes macrocephala Koidz*	PAM	RL95-2 cells	Promote the release of Cyt C in mitochondria to the cytoplasm; Activate the mitochondrial apoptosis signaling pathway; Cause the imbalance between cancer cell Bcl-2 and Bax; Increase Caspase9 enzyme activity; Cleave-Caspase3 activity; Promote tumor cell apoptosis	Yang et al. (2022)
	*Astragalus* polysaccharide	APS	BALB/c nude mice (aged 4–5 weeks, 17.5 ± 2.5 g)	Promote E-cadherin protein and gene expression; Inhibit β-catenin protein and gene expression; Exert anti-tumor effect by regulating Wnt signaling pathway	Qiu et al. (2018b)
	*Lycium barbarum L.* polysaccharide	LBP	BALB/c nu/nu mice	Reduce the damage of immune cells and the inhibition of hematopoietic function by chemotherapy drugs; Promote the recovery of liver and kidney function; Inhibit the occurrence of stress through the Nrf2 signaling pathway	Zhou et al. (2016)
	*Ginkgo biloba exocarp* polysaccharide	GBEP	HEC-1B cells	Inhibit G0/G1 phase to S phase causing cell cycle arrest; Inhibit DNA synthesis	Liu et al. (2012)

The polysaccharide of *Atractylodes macrocephala Koidz.* (PAM) is an active ingredient of Chinese medicine atractylodes. It has proven to have a variety of biological functions, including antitumor, antioxidant, anti-microorganisms, and anti-inflammatory (Zhu et al., 2018). PAM has good immune regulation and apoptosis promotion effects in past studies on colon cancer MC38 cells and gel tumor C6 cells (Li et al., 2014; Feng et al., 2019). Yang et al. (2022) proved that PAM could concentrate and time-dependent *in vitro* experiments to inhibit human uterine endometrial cancer RL95-2 cell proliferation and promote its apoptosis. The dynamic balance between BCL-2 and BAX is the key to controlling apoptosis. Opferman and Kothari (2018) believe that anti-apolipoprotein BCL-2 expresses can inhibit the switching of apoptosis BAX to mitochondria, leading to abnormal cell proliferation. PAM-induced RL95-2 cell apoptosis experiments show that the relative expression of the PAM drug group BCL-2 protein significantly decreases in comparison with the control group cells. In contrast, the expression of BAX and downstream apoptosis executors Cleaved-Caspase3 has increased dramatically, accelerating the RL95-2 cell apoptosis process, suggesting that the mechanism of PAM is related to the activation of mitochondrial apoptosis signaling pathways.

*Astragalus mongholicus Bunge* is an ancient form of TCM, that is, used both in medicine and food. In China, it is known as “Huangq”, considered an excellent gasket by traditional medicine that can improve the human body’s immunity. The main active ingredients of astragalus are *Astragalus* polysaccharide (APS), saponin, alkaloids, and flavonoids. Studies have shown that these substances can effectively improve human immune function (Auyeung et al., 2016; Kong et al., 2021). In animal tests, APS has been proven to have an impressive effect on endometrial cancer. Qiu et al. (2018b) observed the effect of the expression of APS-related factors β-catenin and E-cadherin in the Wnt gene transduction pathway in subcutaneous xenograft tumors of human endometrial cancer in nude mice. Studies have found that the volume and tumor weight of *Astragalus* polysaccharides is significantly lower than the model group. APS can raise the E-cadherin protein and gene expression, inhibit β-catenin protein and gene expression, and play an anti-endometrium cancer effect by regulating the Wnt gene conversion pathway.

*Lycium barbarum L.* is mainly distributed in northern temperate regions such as China, South Korea, Japan, and Europe. Modern medicine believes that wolfberry has biological functions such as antitumor, antioxidant, and immune regulation (Tian et al., 2019). Studies have found that wolfberry polysaccharides can protect against the damage of oxygen free radicals by increasing endogenous hyperoxia enzymes and reducing the horizontal level of propane (Amagase et al., 2009). In a preliminary study, studies such as Zhu and Zhang (2013) have shown that LBP can inhibit the proliferation of tumor cells by changing cell cycle distribution and affecting the synthesis of DNA. At the same time, *in vitro* experiments also showed that LBP could cause the loss of mitochondrial transmembrane potential and the accumulation of intracellular Ca^2+^ in apoptotic cells was also observed by laser scanning confocal microscopy. Interestingly, unlike most polysaccharides, the ability of LBP to inhibit tumor cell proliferation is not dose-dependent, which is also confirmed in research on liver cancer (Zhang et al., 2005). Studies by Zhou et al. (2016) have found that LBP can effectively promote the treatment and extend the average survival time of the treatment and extend the average survival time of the picanol to endometrium cancer. As well as hematopoietic function. The critical problem in researching plant-based drugs is to clarify the substances that play the main biological effects in rough extracts. LBGP-I-3 is a polysaccharide distillation separation from the rough polysaccharides of wolfberry. Gong et al. (2020) found that the antitumor result of LBGP-I-3 was significantly higher than other wolfberry polysaccharides. However, in the manuscripts of Zhou et al., This has not been stated in detail. In addition, the specific mechanism of LBP anti-uterine cancer has been ignored, which makes us only see the rough results.

GBEP is a kind of natural active polysaccharide separated from *Ginkgo biloba endocarp*. Previous studies have shown that GBEP can inhibit tumor proliferation and adjust immune function (Liu et al., 2022). Xu et al. (2003) conducted a clinical study on gast cancer patients with oral GBEP capsules. It was found that GBEP could reduce the area of the tumor and lower C-myc, BCL-2 gene expression, and raise C-fos gene expression to inhibit tumor cell proliferation and induce gastric cancer tumor apoptosis. Liu et al. (2012) used *in vitro* cultivating human endometrium cancer cells HEC-1B to evaluate the characteristics of GBEP inhibiting tumor cell proliferation. Studies have found that the GBEP of 40–160 mg/L presented the proliferation of HEC-1B cells in time and dose-dependence, which can cause increased cell cycle blocking the proportion of cells in the G0/G1 stage and the decrease in the G2/M stage. It is worth noting that although ginkgo is a famous medicinal plant, it is not safe. Endotoxins and ginkgolic acids have been determined to be the main toxic components in different parts of ginkgo. Therefore, the clinical application of Ginkgo derivatives needs to provide all information about the possible interaction risks (Liu et al., 2022).

Although we consulted the literature about natural polysaccharides in anti-uterine endometrial cancer in the past 20 years, unfortunately, whether *in vitro* experiments or *in vitro* experiments, the current research on natural polysaccharides in the application of endometrial cancer is minimal. In our sorting literature, almost all research comes from China. Researchers are more inclined to study Chinese medicines used for thousands of years in the clinic. It should be acknowledged that the development of new drugs for endometrial cancer is far more complicated than cervical cancer and ovarian cancer. The transformation from laboratory to human subjects will be far away (Lin et al., 2014).

### 3.3 Polysaccharides and ovarian cancer

Ovarian cancer is a fatal malignant tumor in all gynecological cancers. Its pathogenesis involves a wide range of molecular mechanisms and genetic modification, making it very difficult to treat (Karnezis et al., 2017). Globally, due to the lack of the initial symptoms of ovarian cancer and the deficiency of screening methods, most of the patients have entered the advanced stage of the disease at the clinical diagnosis, and the 5-year survival rate is only 47% (O'Connell et al., 2022; Xu and Cao, 2022). During the treatment of patients with advanced ovarian and recurrent ovarian cancer, the use of first-line chemotherapy, which is usually a combination of karplatinism or cispiene and paclitaxel compounds after tumor reduction, is the preferred plan for patients. Unfortunately, almost all patients with recurrent ovarian cancer will eventually develop platinum resistance (Tabury et al., 2022). Natural polysaccharides not only intervene in inflammation and immune response, regulate tumor cell cycles, affect cell migration, and invade anti-ovarian tumors, but also have a safe and effective chemotherapy sensitivity, having good development potential in the treatment of ovarian cancer. In Table 3, we list the detailed results of natural polysaccharides anti ovarian cancer.

**TABLE 3:** Compilation of experimental data related to the protective effect of natural polysaccharides anti-ovarian cancer.

Classification	Compound/Extract	Abbreviation of polysaccharides	Cell Line/Animal model	Mechanism and effect	References
Polysaccharides of higher plants	*Pyracantha fortuneana* polysaccharide	PSPF	SKOV3 cells	Increase ROS levels; Induce nuclear DNA rupture, leading to mitochondrial destruction and DNA damage	Yao et al. (2020)
	Selenium-enriched polysaccharides from *Pyracantha fortuneana*	Se-PFPs	HEY and SK-OV-3 cells; BALB/C nude mice (aged 30 days)	Suppress β-catenin signaling in a GSK-3β–dependent mechanism; Inhibit EMT change	Sun et al. (2016)
	*Astragalus* polysaccharide	APS	OV90 cells, SK-OV-3 cells	Eliminate the inhibitory effect of miR-27a on FBXW7 to play an anti-tumor role; Reduce the expression of miR-27a and cause the upward adjustment of the tumor inhibitor gene FBXW7, inhibiting cell proliferation in ovarian cancer cells and promote cell apoptosis	Guo et al. (2020)
			SKOV3 cells	Decrease the expression of Bcl2; Activate the JNK pathway to increase the sensitivity of SK-OV-3 cells to cisplatin	Li et al. (2018b)
	*Menispermum dauricum* polysaccharide	MDP-A1 and MDP-A2	SKOV3 cells; BALB/C nude mice	Reduce the expression of caspase-3 and caspase-8; Induce apoptosis	Lin et al. (2013)
	*Polygala tenuifolia* polysaccharide	PTP	SKOV3 cells; BALB/c nude mice (aged 6–8 weeks, 21.0 ± 2.0 g)	Downregulate protein and mRNA levels of EGFR, VEGF and CD34; Interfere with the NF-κB pathway in SK-OV-3 cells; Lower the activity of Bmi-1 and telomerase; Induce apoptosis	Yao et al. (2018)
	*Balanophora polyandra* polysaccharide	BPP	A2780cells, OVSAHO cells; BALB/c nu/nu mice (approximately aged 5 weeks, 16 g)	Increase the expression level of Bax; Reduce the expression level of Bcl-2; Increase the expression of p53; lower the expression of p21 gene; Inhibit CDK activity	Qu et al. (2020)
	*Portulaca oleracea L.* polysaccharide	PSP	Wistar rats (aged 40 days, 200–250 g)	Scavenge free radical activity; Protect oxidative damage; Enhance thymocyte proliferation in a concentration-dependent manner	YouGuo et al. (2009)
	*Basil* polysaccharide	BPS	SK-OV-3 cells	Downregulate of OPN and MMP-9 expression to inhibit invasion of SK-OV-3 cells and DCs	Lv et al. (2013)
Polysaccharides from fungi	*Pleurotus djamor* polysaccharide	PS	PA1 cells	Inhibit cell proliferation	Maity et al. (2019)
	β-glucan extracted from *Agaricus blazei Murill*	/	HRA cells; inbred nude (BALB/c nu/nu) mice	Improve the activity of p38 MAPK; Inhibit the proliferation; Amplify the cascade reaction of apoptosis; Induce Bax translocation, cytochrome C release, and caspase-9 activation	Kobayashi et al. (2005)
	*Ganoderma lucidum* polysaccharide	/	OVCAR-3 cells	Down-regulat cyclin D1 inhibits cell growth; Disrupt cell cycle progression	Hsieh and Wu (2011)
Polysaccharides from algae	*Bangia fuscopurpurea* polysaccharides	BFP	A2780 cells	Increase the production of ROS; Reduce MMP; Increase the expression of Bax and BAX/BCL2 ratio; Reduce the expression of Bcl-2; Promote cell apoptosis; Increase the expression of Beclin-1; Reduce the expression of P62; Promote cell autophagy	Wu et al. (2021)
	Fucoidan	Fucoidan	ES2cells, OV90 cells	Initiate depolarization of MMP, ROS production; Increase the calcium ion concentration in cytosol and mitochondria; Inhibit PI3K/MAPK intracellular signaling pathway; Activate apoptosis cascade and ER stress sensor protein; Decrease mRNA expression of VEGFs (VEGFA-VEGFD); Exert anti-angiogenesis effect	Bae et al. (2020)
	Marine microalgal exopolysaccharide	EPS	BG1 cells	Inhibit the protein expression of cell cycle-related genes cyclin D1 and cyclin E; Improve immune response by increasing B cell proliferation and antibody production	Park et al. (2017)
Polysaccharides from animals	*Puried C. fluminea* polysaccharides of molecular weight 22 kDa	CFPS-2	SK-OV-3 cells, A2780 cells	Scavenge activity on free radicals, such as superoxide radicals and hydroxyl radicals	Liao et al. (2013)

#### 3.3.1 Polysaccharides of higher plants

*Pyracantha fortuneana* (Maxim.) Li, a plant species of Maloideae, is widely used in the biomedical field and has been used as an excellent TCM for the treatment of abdominal distension (Peng et al., 2016). Pyracantha fortune can play antioxidant and immune protection in animal experiments (Yuan et al., 2015). Yuan et al. (2015) used response surface methodology to prove the antitumor effect of polysaccharides from Pyracantha fortune (PSPF).In this study, researchers used a fluorescent probe DCFH-DA to detect the ROS levels in SK-OV-3 cells, they found that the fluorescence intensity of DCF in the PSPF treatment group was significantly higher than that in the control group, suggesting that PSPF can induce the increased of ROS levels in SK-OV-3 cells, leading to mitochondrial destruction and DNA damage. And then play a cytotoxic effect. In terms of cell morphology, the shape of the SK-OV-3 cells in the treatment group is transformed from the previous slender spindle-shaped body to a circular shape after 200 μg/ml PSPF treatment, which also indicates that PSPF promotes the apoptosis of tumor cells. Another new type of selenium-rich polysaccharide, Se-PFPs, discovered in recent years from Pyracantha fortune, has also been confirmed to suppress the β-catenin signal pathway and epithelial-mesenchymal transition through *in vitro* and *in vivo* experiments (Liu et al., 2014; Sun et al., 2016). Therefore, PSPF and Se-PFPs can be used as functional dietary additives to become clinical treatment agents for ovarian cancer patients (Sanmartin et al., 2012).

*Astragalus* polysaccharide (APS) is a naturally new type of antitumor polysaccharide. Previous studies have confirmed that it can induce the apoptosis of nasopharyngeal carcinoma cells and osteosarcoma cells (Zhou et al., 2017; Chu et al., 2018) and shows the role of inhibiting tumor growth in an animal experiment (Zhou et al., 2018). Guo et al. (2020) reported the part and mechanism of APS inhibiting ovarian cancer cell growth. Microarray assay. It was found that APS could reduce the proliferation of ovarian cancer cells and induce them to apoptosis. The specific mechanism of invasion and migration is achieved by reducing the expression of the MIR-27A and raising the tumor inhibitory gene FBXW7. The BAX and BCL-2 genes are currently crucial in controlling apoptosis in the BCL-2 family (Garrido et al., 2006). While Li et al. (2018b) found that ASP weakened the survival of SK-OV-3 cells, it can increase the expression of BAX and caspase-3 and activate the JNK1/2 signaling pathway by lowering the expression of BCL2, thereby increasing the sensitivity of ovarian cancer to cisplatin treatment. Therefore, APS has the potential to develop a new sensitive agent for ovarian cancer.

MDP-A1 and MDP-A2 are two kinds of natural polysaccharides extracted from the rhizomes of *Menispermumum dauricum*. Unlike previously studied antitumor polysaccharides, MDP-A1 and MDP-A2 are acidic, filling the blank of natural acidic polysaccharides in ovarian cancer research. Lin et al. (2013) discovered that MDP-A1 and MDP-A2 can not only significantly inhibit the proliferation of SK-OV-3 cells *in vitro*, but the effect of antitumor is also confirmed in the BALB/c nude body of the injection of SK-OV-3 cells. MDP-A1 and MDP-A2 can reduce the expression of caspase-3 and caspase-8 and induce the apoptosis of ovarian cancer cells. Interestingly, MDP-A1 and MDP-A2 have the same sugar composition both of which are composed of glucose, mannose, galactose, arabic sugar, glucosol acid, and lactic acid. There are certain differences in molar ratio, and such a slight gap also makes the two slightly different in anti-tumor activity. MDP-A2 shows a better antitumor effect than MDP-A1, but the detailed mechanism of the difference in effects does not explicitly clarify and still needs to be discussed.

*Polygala tenuifolia* polysaccharide (PTP) is a novel natural water-soluble natural polysaccharide separated from Chinese medicine *Polygala tenuifolia L.*, which has been reported to suppress the growth of ovarian cancer *in vitro* and body effectively. Yao et al. (2018) tested different doses of PTP’s ovarian tumor volume changes in BALB/c nude mice. The results showed that the tumor volume of the mouse model was dropped from 868.78 mm^3^ of the unbearable group to 40 mg/kg of the dose of 40 mg/kg. At the time of 214.45 mm^3^, this inhibitory phenomenon is concentrated dependence (Xin et al., 2012). The study also confirmed that PTP was to lower the vascular endothelial growth factor (VEGF), the epidermal growth factor receptor (EGFR), and the protein and mRNA levels of CD34, inhibiting the formation of tumor new vascular formation. In addition, studies have found that PTP can interfere with the NF-κB pathway in SK-OV-3 cells and reduce Bmi-1 and telomerase activity (Zhang et al., 2015a; Zhang et al., 2015b). These studies show that PTP’s mechanism for inhibiting ovarian cancer is complicated and has massive potential in regulating immunity and controlling inflammation reactions.

BPP is a kind of natural polysaccharide obtained from *Balanophora polyandra Griff.* Qu et al. (2020) discovered BPP to inhibit the proliferation of ovarian cancer cells in time and dose-dependent ways. This mechanism is related to the induction of cycle arrest in A2780 and OVSAHO cells by reducing the expression of cyclin-dependent kinase 2 (CDK2). P53, as a carcinogenic suppression gene to control the integrity of the genome, plays a vital role in cell damage repair (Goodheart et al., 2005). In this study, the researchers also found that the BPP could inhibit the proliferation of ovarian cancer by affecting the P53 signaling pathway. The manuscript does not detail the relationship between the BPP and the P53 signaling pathway. However, this study still shows that BPP can be a potential therapeutic agent for ovarian cancer therapy.

*Ocimum Basilicum L.* is native to Southeast Asia and parts of the Americas. It has good antitumor, antioxidant, immune regulation, blood sugar, and antibacterial effects (Uma Devi, 2001). BPS is a naturally active polysaccharide extracted from Lawler. Studies have found that it has an excellent dynamic cancer effect. Lv et al. (2013) treated SK-OV-3 cells and DCS with 100 μg/ml BPS and found that BPS can reduce the invasion of SK-OV-3 cells and the MMP-9 expression of mRNA and protein in SK-OV-3 cells. This study supports BPS as an effective supplementary therapy for ovarian cancer.

#### 3.3.2 Polysaccharides from fungi

*Agaricus blazei Murill* is a type of Basidiomycete fungus. The β-glucan separated from the water-soluble part of its extract is a potential antitumor polysaccharide. Kobayashi et al. (2005) through internal and *in vitro* experiments found that β-glucan can enhance P38 MAPK activity, stimulate the optimal protein BAX to mitochondria, and then activate caspase-9 to inhibit HRA cell proliferation. The study found that in the disseminated metastatic model of nude mice ovarian cancer, a certain amount of tumor load related to the naked ovarian cancer peritoneal metastasis protected by a certain amount of β-glucan is significantly reduced daily. Interestingly, the inhibitory of β-glucan in the tumor is divided. This experiment shows that *in vitro* experiments, β-glucan does not have inhibitory effects on Lewis lung cancer 3LL cells. Maity et al. (2019) separated a water-insoluble β-glucan from the edible bacteria *Pleurotus djamorjammer*. After research, when the β-glucan concentration reaches 250 μg/ml, almost all ovarian cancer PA1 cells are destroyed, revealing its significant anti-resistance cancer effect. In addition, the natural polysaccharides in *Ganoderma lucidum* can also be confirmed that they can regulate the growth of OVCAR-3 cell lines and destroy the cycle process of the cell by lowering cyclin D1 (Hsieh and Wu, 2011). Overall, β-glucans isolated from different fungi have potential anticancer effects, and such polysaccharides may be more beneficial in the clinic for ovarian cancer patients with metastasis or at risk of metastasis.

#### 3.3.3 Polysaccharides from algae

*Bangia fuscopurpurea* polysaccharides are natural polysaccharides separated from *Bangia fuscopurpurea* (BFP). Wu et al. (2021) proved that the A2780 cells processed by BFP showed a dose-dependent increase in LC3 levels. At the same time, the essential protein BeClin-1 expression of the autophagy body is increased, which indicates that the accumulation of autophagosome formation increased after BFP intervention, while the number of autophagosomes did not change in untreated A2780 cells. Hseu et al. (2019), Feng et al. (2020) researchers found that the accumulation of ROS in cells can induce cells to develop in the direction of apoptosis. When using ROS inhibitors NAC and different concentrations of BFP processing A2780 cells, the formation of ROS can decrease as the BFP concentration increases, and there are calculated changes in the apoptosis rate, which indicates that the BFP can promote ROS accumulation, and then induces the apoptosis of A2780 cells. In addition, the study also found that BFP activated the mitochondrial apoptosis pathway in A2780 cells, induced the decrease of mitochondrial membrane potential in A2780 cells, and then led to the activation of caspase-9 and the release of cytochrome C (Yu et al., 2020). Another polysaccharide, Fucoidan, separated from Brown Algae, has been confirmed by researchers it also has an anti-OC role. Its mechanism to inhibit the proliferation of ES2 and OV90 cells is related to regulating ER stress and calcium level (Bae et al., 2020).

Marine microalgal exopolysaccharide (EPS) is a polysaccharide from marine microalgae *Thraustochytriidae sp*. It is reported that it has excellent potential in antioxidant, anti-proliferation, antiviral, and immune regulation and has attracted the attention of scientists (Xiao and Zheng, 2016). To study and verify the potential antitumor cell proliferation and immune regulation of EPS on OC, Park et al. (2017) performed *in vitro* experiments on BG-1 cells. They found that EPS can inhibit the protein expression of CyClin D1 and CyClin E-related genes related to cell cycles, thereby promoting the apoptosis of ovarian cancer cells. Specifically, the reduction of cyclin D1 and cyclin E directly interrupted the cell cycle process and cell division of OC cells, which prevented the transition from phase G1 to S (Baldin et al., 1993; Donnellan and Chetty, 1999). In addition, in terms of the immune response, research also revealed that this process is related to EPS increasing B cell proliferation and antibody production. These results show that marine algae have the potential for experiments, indicating that Marine algae have the potential to be developed as a functional food for ovarian cancer therapy.

#### 3.3.4 Polysaccharides from animals

CFPS-2, a polysaccharide extracted from a freshwater clam, *Corbicula fluminea*, can regulate the function of immune cells and lower cholesterol. *Corbicula fluminea* has been a dual-use medicinal herb since ancient times. It is a TCM against various diseases. In terms of tumor cell formation and malignant transformation, Devi et al. (2000) reported that free radical generation could accelerate oxidation stress damage, causing lipid peroxidation and mutations in DNA (Zhou et al., 2004). Liao et al. (2013) found that CFPS-2 could significantly inhibit the growth of SK-OV-3 cells and A2780 cells *in vitro*. Hydroxyl radicals and superoxide radicals contained in CFPS-2 are natural antioxidants. Their anti-ovarian cancer effect is related to the high content of sulfate radicals in CFPS-2 in the structure, limiting the damage caused by reactive oxygen species. It should be noted that animal polysaccharides are more likely to cause allergic reactions, which makes us need to be cautious when using animal polysaccharides, especially in certain conditions and particular populations (Lei and Grammer, 2019).

## 4 Novel formulations of polysaccharides in the treatment of gynecological cancers

With the development of technology, we can find the parts or methods with the highest polysaccharides from the raw materials, as Liu et al. (2019) used the DEAE-Berpharose FF ion exchange chromatography to separate the components of crude polysaccharide and crude polysaccharide complex extracted from green fruit. It was found that the total polysaccharide content in the eluted part of distilled water solution was the highest, and the inhibitory effect on the proliferation of human cervical cancer HeLa cells was the strongest. Even so, it is still difficult to avoid the problem of low polysaccharide biological utilization rate. To improve the treatment potential of polysaccharides, new dosage types and technologies such as microspheres, microcapsules, liposomes, and nanoparticles have gradually become a new direction for polysaccharides preparation research.

Since the 1960s, the study of microcapsules has begun to rise. In recent years, the drug system for microcapsules as a polysaccharide has gradually attracted people’s attention. Shu et al. (2014) prepared microcapsules of Schisandschisandra polysaccharide by spray drying method with chitosan as a carrier. The experimental results showed that the structure of Schisandschandra polysaccharide was not damaged by this preparation. In the early release stage, according to the difference in concentration, the pharmaceutical molecule spreads quickly from the micropores to form an emergence effect. After that, the capsule was absorbed and swollen, and the micro-hole diameter decreased, which played a slow-release effect. The fairy palm polysaccharide (Yin et al., 2019b), which has been proven to have anti-ovarian cancer cell proliferation effects, is prepared to prepare the actual polysacchaser macro deck after using the emulsification-cross-linking method only improves the capacity and packaging rate of the load but also increases the stability of polysaccharides.

In recent years, the popular study of nanoparticles is due to their unique physical chemistry and biological characteristics, making it easier to pass through the blood vessels into the body circulation through blood vessels. It is conducive to the absorption of biological microtomes such as polysaccharides (Zheng et al., 2010). Li et al. (2010b) studied the *ganoderma lucidum* polysaccharide nanoparticles preparation, the ion gel method is used to convert the *ganoderma lucidum* polysaccharide package within the chitosan carrier material. Its experimental results show that compared with the direct use of *ganoderma lucidum* polysaccharide, polysaccharide nanoparticles significantly increase the antitumor activity of *ganoderma lucidum* polysaccharides, promoting the growth of the spleen cells, with a stimulative effect on lymphocytes enhancing immunity. The size of the nano-emulsion preparation is smaller, which not only improves the solubility of insoluble drugs but also protects unstable and easily degradable drugs and improves the stability of drugs.

In addition, Kong et al. (2020) prepared low molecular weight enzyme hydrolyzed *ganoderma lucidum* polysaccharide by cellulase hydrolysis method and confirmed its anti-tumor effect on cervical cancer U14 mice. Since the 1970s, lipids have been used as drug carriers by Rahmen and others. It has attracted people’s attention because of its simple preparation, non-toxic to the human body, non-immunity, easy to achieve, and so on. Wu et al. (2011) separated liposomes and free actinidia planch polysaccharide by anion exchange resin method, ultraviolet-visible spectrophotometry to determine the content of free actinidia planch polysaccharide, and calculate the sealing rate of liposomes. Oral administration of kiwifruit root polysaccharide has a good antitumor effect and can extend the circulation time *in vivo*. Liposomes coated with ophiopogonis polysaccharide showed significantly enhanced immunoenhancing activity, but the role of these liposomes in gynecological cancers needs to be further verified (Liberato et al., 2013).

Moreover, studies have shown that combining polysaccharides and some active components may exert more potent anticancer efficacy. For example, the spirulina polysaccharides and active members of Ginkgo biloba left at different doses can exert more potent inhibitory effects on human cervical cancer HeLa cells (Hao et al., 2009). In addition, the polysaccharide is also regarded as a drug-carrying structure and plays a role in carrying antitumor drugs (Akasov et al., 2015; Dheer et al., 2017). Deng et al. (2016) reported the design of a simple hybrid microcapsule composed of polysaccharides (sodium alginate, chitosan, and hyaluronic acid), iron oxide, and graphene oxide. The polysaccharide component ensures higher biocompatibility, bioavailability, and tumor cell targeting of anticancer drugs. The anticancer potential and efficiency of polysaccharides can be significantly improved by improving the formulation of natural polysaccharides and utilizing new drug delivery systems. However, this study is rare in gynecological cancers, and more experimental studies and clinical trials are needed to confirm the efficacy of new polysaccharide dosage forms in treating gynecological cancers.

## 5 Conclusion

From the foregoing review, the role of natural products in gynecological cancer treatment is constantly well-known, and its clinical value continues to be valued. Research on natural products has gone deep into improving chemotherapy drugs and tumor immunotherapy (Xu et al., 2021). The diversity of polysaccharides depends mainly on their plants or biological sources, and their unique structure helps them successfully apply in the field of gynecological cancers.

What is noteworthy is that the current research on plants is still mainly based on plant polysaccharides, which may be closely related to people’s application of these traditional herbs for thousands of years. Previous studies have found that the mechanism of natural product intervention in gynecological cancers is mainly associated with cytotoxic effects, inhibiting proliferation and promotion of apoptosis, cell migration, reactive oxygen injuries, induced autophagy cell death, inhibiting vascular production, and promoting DNA damage (Deng et al., 2016). Our research found that polysaccharide anti-gynecological cancers are consistent with that other cancers such as lung or colorectal cancer, etc. Meanwhile, our research found that in cervical cancer and endometrial cancer, more polysaccharide can improve the immune organ index, reduce the generation of free radicals to protect against oxidative damage, and regulate the expression level of immune molecules and so on to protect the original function of the body.

However, we must realize that the research on fungal polysaccharides, algal polysaccharides, and animal polysaccharides in gynecological cancer is still insufficient. According to the latest evidence of natural polysaccharides in the treatment of gynecological tumors, there are still many questions that need to be answered.

1)  Although more studies have shown that natural polysaccharides are effective in the treatment of various tumors, most studies are limited to cell and animal experiments, and there are relatively few clinical studies on the effectiveness of natural polysaccharides. It is worth mentioning that due to the complexity of polysaccharide structure, most studies are limited to the biological activity mechanism of polysaccharides, but the relationship with polysaccharide structure is ignored. What is more important is the clinical application of natural polysaccharides. At present, most plant polysaccharides have not been widely used in clinical preparations, while fungal polysaccharides are relatively widely used, which may be related to the difference in molecular structure. At present, the study of polysaccharide compounds in drug toxicology still needs a lot of research to supplement the effective evidence of its clinical application, so there are still many challenges to the use of natural polysaccharides in the clinical treatment of gynecological tumors.
2)  In addition, it is necessary to point out that although there are many types of natural polysaccharides, the low degree of biological utilization of polysaccharides is still a problem we have to face. Focusing on the structure-activity relationship of polysaccharides to make polysaccharide derivatives more effective is also a new way to improve the bioavailability of polysaccharides. To improve the bioavailability, it not only includes the content of polysaccharide extraction source but more importantly, in the process of future clinical application, the molecular structure, molecular weight, functional group, spatial conformation, main chain, and branched chain of polysaccharide are modified to make its activity behave like *in vitro* experiments.
3)  We searched the latest literature evidence and found that there is still a lack of research on the specific components and targets of natural polysaccharides in the treatment of gynecological tumors. In the future, further pharmacokinetic experiments are needed to prove the absorption, distribution, metabolism, and process of natural polysaccharides after entering the body, to further clarify the trend of biological functions of polysaccharides.
4)  The key to natural polysaccharides as drugs lies in their complex molecular structure, multiple biological functions, and multiple molecular targets. Although the current evidence shows that toxic side effects caused by natural polysaccharides are rare, it is important to determine the dose-response relationship for the treatment of gynecological tumors. Different doses and drug components may produce different anti-cancer effects and protective effects on the female reproductive system. How to standardize the clinical application dose of polysaccharides and how to conduct a reliable polysaccharide dose-response relationship study will be the problems to be solved in the future.
5)  Coincidentally, contemporary life sciences, represented by molecular biology, have also entered the field of non-intuitive horizontal life matter research, and have developed a variety of emerging disciplines such as structural biology, network biology, and systems biology. Some studies began to pay attention to the direction of life material groups and formed a complete set of life material analysis and testing technology. Using these technical means, we can organize the chain and conformation to form a database on the anti-gynecological tumor research of natural polysaccharides, integrate the structural heterogeneity and function of polysaccharides as much as possible, and make it easy to obtain and analyze, to fully understand the natural The overall mechanism of tumor cells affected by polysaccharide therapy and its compounds.

In addition, many polysaccharides still stay in easy use of cancer cells or mouse models for tests. So far, no high-quality clinical trial data can be used to evaluate the effectiveness of natural polysaccharides in cancer patients. In the future, the direction of hard work should be carefully designed to verify the actual clinical efficacy of natural polysaccharides. We believe that in the future, natural polysaccharides will be an essential direction for the development of new tumors, and their detailed molecular mechanism will also be more precise explanations.

## Figures and Tables

**Table:** 

**LNT**	Lentinan
**HPV**	human papilloma virus
**ROS**	Reactive oxygen species
**EMT**	Epithelial mesenchymal transition
**POL-P3b**	Portulaca oleracea L. polysaccharide
**DC**	dendritic cell
**U14**	Human cervical cancer U14 cells line
**SNL-P**	Solanum nigrum L. polysaccharide
**PHB-P1**	Patrinia heterophylla Bunge polysaccharide
**ACPS-1**	Atractylodes lancea (Thunb.) DC. polysaccharide
**HeLa**	Human cervical cancer HeLa cells line
**SK-OV-3**	Human ovarian cancer SK-OV-3 cells line
**GC**	Garden Cress
**GCP**	Garden cress polysaccharide
**ASPS**	Acanthopanax senticosus polysaccharide
**APS-1d**	Angelica sinensis (Oliv.) Diels polysaccharide
**HPA**	Hansfordia sinuosae extracellular polysaccharide
**EGLP**	Enzymatic hydrolysis of Ganoderma lucidum polysaccharide
**PRG1-1**	Polysaccharide extracted from wild Russula griseocarnosa
**LEP1**	Lentinus edodes polysaccharide
**EPS**	Extracellular polymeric substances
**LJSP**	Laminaria japonica sulfate polysaccharide
**RL95-2**	Human endometrial cancer RL95-2 cells line
**HEC-1B**	Human endometrial cancer HEC-1B cells line
**PAM**	polysaccharide of Atractylodes macrocephala Koidz
**APS**	Astragalus polysaccharide
**LBP**	Lycium barbarum L. polysaccharide
**GBEP**	Ginkgo biloba exocarp polysaccharide
**PSPF**	Pyracantha fortuneana polysaccharide
**Se-PFPs**	Selenium-enriched polysaccharide from Pyracantha fortuneana
**HEY**	Human ovarian cancer HEY cells line
**BG1**	Human ovarian cancer BG1 cells line
**OV90**	Human ovarian cancer OV90 cells line
**PA1**	Human ovarian cancer PA1 cells line
**ES2**	Human ovarian cancer ES2 cells line
**A2780**	Human ovarian cancer A2780 cells line
**MDP**	Menispermum dauricum polysaccharide
**PTP**	Polygala tenuifolia polysaccharide
**BPP**	Balanophora polyandra polysaccharide
**BPS**	Basil polysaccharide
**MMP**	Matrix metalloproteinase
**PSP**	Portulaca oleracea L. polysaccharide
**PS**	Pleurotus djamor polysaccharide
**BFP**	Bangia fuscopurpurea polysaccharide
**EPS**	Marine microalgal exopolysaccharides
**CFPS-2**	Puried Cfluminea polysaccharides of molecular weight 22 kDa
**ER**	endoplasmic reticulum
